# Single dose oral ranolazine pharmacokinetics in patients receiving maintenance hemodialysis

**DOI:** 10.1080/0886022X.2019.1585371

**Published:** 2019-03-26

**Authors:** Bridget A. Scoville, Jonathan H. Segal, Noha N. Salama, Michael Heung, Barry E. Bleske, Rachel F. Eyler, Bruce A. Mueller

**Affiliations:** a Saint Alexius Medical Center, Hoffman Estates, IL, USA;; b Department of Internal Medicine, University of Michigan School of Medicine, Ann Arbor, MI, USA;; c Department of Pharmaceutics and Industrial Pharmacy, Faculty of Pharmacy, Cairo University, Cairo, Egypt;; d Department of Pharmaceutical and Administrative Sciences, St. Louis College of Pharmacy, St. Louis, MO, USA;; e Department of Pharmacy Practice and Administrative Sciences, University of New Mexico College of Pharmacy, Albuquerque, NM, USA;; f Department of Pharmacy Practice, University of Connecticut School of Pharmacy, Storrs, CT, USA;; g Department of Clinical Pharmacy, University of Michigan College of Pharmacy, Ann Arbor, MI, USA

**Keywords:** Ranolazine, hemodialysis, pharmacokinetics, end stage renal disease

## Abstract

**Purpose:** Ranolazine is a novel anti-angina treatment approved in the United States for chronic stable angina. Ranolazine pharmacokinetics have not been studied previously in patients who receive maintenance hemodialysis. This study describes the pharmacokinetics of ranolazine and three major metabolites (CVT-2738, CVT-2512, CVT-2514) in patients receiving thrice weekly hemodialysis.

**Methods**: Eight participants receiving maintenance hemodialysis completed this prospective, open-label study (study identifier NCT01435174 at Clinicaltrials.gov). Three participants received a single tablet of ranolazine 500 mg (followed by an interim analysis), and five received 2 tablets of ranolazine 500 mg. Blood samples were collected over 65 h to determine the pharmacokinetic characteristics during and between hemodialysis sessions. Non-compartmental analysis was used to determine the individual pharmacokinetic parameters.

**Results:** Ranolazine off-hemodialysis elimination phase half-lives were 3.6 and 3.9 h for 500 mg and 1000 mg doses, respectively. The time to maximum concentration ranged from 2 to 18 hours and the average maximum concentration was 0.65 ± 0.27 mcg/mL and 1.18 ± 0.48 mcg/mL for ranolazine 500 mg and 1000 mg dose, respectively. The mean hemodialysis percent reduction ratio for the ranolazine 500 mg dose was 52.3 ± 8.1% and for the ranolazine 1000 mg dose was 69.2 ± 37.6%.

**Conclusions:** Data on ranolazine dosing in patients receiving maintenance hemodialysis is almost non-existent. Given the extent of pharmacokinetic variability observed with the 500 mg and 1000 mg oral doses of ranolazine, neither can be recommended as a starting dose in patients receiving maintenance hemodialysis. Guided by the information gained form this study about the extent of hemodialytic drug clearance, further multi-dose clinical trials of ranolazine are needed to optimize therapeutic outcomes in this patient population.

## Introduction

Patients with end stage renal disease (ESRD) receiving maintenance hemodialysis have many co-morbidities including unstable angina. In fact, cardiovascular disease is one of the leading causes of death in patients receiving hemodialysis [1,2]. A review by Agrawal et al. discussed the efficacy and safety of different therapies for acute and chronic coronary heart disease in patients with advanced chronic kidney disease (CKD) with emphasis on those receiving hemodialysis and concluded that cardiovascular disease accounts for 45–50% of deaths in patients receiving dialysis [3]. A publication based on the MERLIN-TIMI 36 highlighted the correlation of the decline in renal function with increase in cardiovascular risk factors, decrease in evidence based medical interventions and poor clinical outcomes on the short and long term [4]. The latest updates by the United States Renal Data System (USRDS), the largest and most comprehensive national ESRD and CKD surveillance system established in 1989, confirm that cardiovascular diseases account for over half the deaths with known causes for ESRD patients [5]. Clinical updates from Kidney Disease: Improving Global Outcomes (KDIGO) suggest that published data regarding drug dosing for patients receiving any form of renal replacement therapy is inadequate [6]. Aside from little to no renal elimination, the volume of distribution, protein binding, and non-renal clearance in this patient population are altered compared to patients without kidney disease [6]. These altered pharmacokinetics make it challenging to dose medications in patients receiving maintenance hemodialysis. The treatment of cardiovascular diseases in this population can be difficult as patients receiving hemodialysis are at risk of hypotension and other hemodynamic changes during a hemodialysis session. Therefore, clinicians limit the use of medications before hemodialysis that further potentiate hemodynamic instability.

Unlike current first line agents for chronic unstable angina, such as beta blockers and calcium channel blockers, ranolazine has been proven to have little to no effect on heart rate or blood pressure [7]. However, the short half-life of the original immediate release tablet compromised its clinical utility and led to the development of an extended release formulation [8]. In the United States, ranolazine has been approved for the treatment of chronic stable angina [9]. Age and bodyweight were reported to have significant effects on ranolazine pharmacokinetics but there was no effect for sex [10]. Ranolazine is extensively metabolized by the liver CYP3A and 2D6 enzymes. The three major metabolites are CVT-2512 (produced by dearylation), CVT-2514 (produced by O-demethylation) and CVT-2738 (produced by N-dealkylation) [10].

The maximum plasma drug concentration (*C_max_*) for ranolazine is reportedly high in patients with renal failure independent of the degree of impairment [9]. Hence, the drug does not need to be adjusted in CKD stages 1–4, but the extent with which the drug is removed by hemodialysis (ESRD) remains unknown [9] and reports have suggested that dose reduction may be required [3]. A pharmacokinetic study in subjects with severe renal impairment was stopped because of acute renal failure after 500 mg twice daily for 5 days followed by 1000 mg twice a day [9]. Based on ranolazine’s low molecular weight of 428 Daltons, one would predict ranolazine to be efficiently removed during hemodialysis, but ranolazine’s relatively large volume of distribution (85–180 L) and moderate amount of protein binding (61–64%) suggest less efficient hemodialysis clearance. The complexity of these pharmacokinetic properties make it difficult to predict the extent with which ranolazine will be removed by hemodialysis. *To date, there are no studies that discuss ranolazine pharmacokinetics in hemodialysis patients*.

One publication in 2005 explored the pharmacokinetics of ranolazine and its 3 major metabolites in patients with renal impairment and revealed a significant decrease in the renal clearance for the drug and its metabolites all renally impaired subjects when compared to healthy volunteers [11]. However, similar to the MERLIN-TIMI 36 trial that was referenced above, patients on hemodialysis were excluded from the trial. A decade after the FDA’s approval of ranolazine (Ranexa), drug dosing in patients receiving hemodialysis remains a challenge and clinicians are left to adopt the old trial and error approach with no insight as to the extent of ranolazine removed by hemodialysis. The primary objective of this pilot study was to characterize the pharmacokinetics of ranolazine in patients undergoing hemodialysis.

## Methods

### Subjects

The study was approved by the University of Michigan Institutional Review Board and conformed to guidelines for ethical conduct of clinical research as outlined in the Declaration of Helsinki. Our study design was based on Jerling and Abdallah’s publication discussing ranolazine pharmacokinetics in patients with renal impairment [11]. We aimed to explore the drug’s pharmacokinetics in hemodialysis patients and compare our findings to the published data regarding patients with varying degrees of renal function and healthy volunteers.

Patients aged 18–74 with a diagnosis of ESRD receiving three times weekly maintenance hemodialysis for at least 3 months were considered for study inclusion. Patients had to weigh >40 kg, be within 50–150% of their ideal body weight, have an estimated glomerular filtration rate of <10 mL/min, have no evidence of infection, and be able to give informed consent. Patients were excluded if they had a QTc interval >440 msec within the last 6 months, pre-study hemoglobin <10 g/dL, plasma albumin <2.5 g/dL, liver disease with a Child Pugh score of C or higher, hepatitis B infection, unstable blood pressure control, a routine need for >4 L of fluid removal during hemodialysis, concomitant use of drugs that were QT-prolonging, major P-gp inhibitors, CYP3A4 inducers, or CYP3A4 inhibitors, a positive pregnancy test or were breastfeeding, documented allergy to ranolazine, or were participating in another investigational drug study. Investigators prescreened potential study participants. Those who met the inclusion and exclusion criteria were invited to participate, and informed consent was obtained. If not performed within the last 60 days, all participants received a physical exam and laboratory tests including hemoglobin, albumin and a 12-lead electrocardiogram. Female participants of childbearing potential had a serum beta-HCG pregnancy test within 14 days before receiving the study drug.

### Study design

This was a prospective open-label investigator-initiated clinical trial. Participants were recruited from the University of Michigan outpatient hemodialysis units. In the absence of any information regarding the extent of drug lost by hemodialysis, it was decided *apriori* that an interim analysis would be performed after 3 participants receiving a single dose completed the study to ensure detectable concentrations of ranolazine and make any necessary study design adjustments accordingly. After analyzing the results from the 3 patient-interim analysis and following medical consultation, the exclusion criteria were modified to exclude patients with a QTc interval >470 msec within the last 6 months and a hemoglobin concentration <9.5 g/dL.

Participants were admitted into the Michigan Clinical Research Unit the day prior to their typical hemodialysis session day. Food was reported to not have any important effect on the *C_max_* and area under the plasma drug concentration curve (*AUC*) of ranolazine [9]. Therefore, participants enrolled prior to the interim analysis were administered a single tablet of ranolazine extended release formulation of 500 mg, non-fasting, 18 h before dialysis. Plasma samples (4 mL) were collected at time 0 (pre-dose), 1, 2, 4, 8, 12, 15, 18, 20, 22, 23, 30 and 65 h post-dose. The interim analysis revealed that drug concentrations following a single 500 mg dose were close/below the limit of quantification after 15 h (not reflected in the figures). Hence, the dose was increased to 1000 mg, administered as two 500 mg-tablets of ranolazine extended release formulation, with the participant fasting 2 h before and after administration. Further, the plasma sampling scheme was adjusted to 0 (pre-dose), 1, 4, 8, 12, 15, 18, 20, 22, 23, 26, 30, 65 h post-dose to provide sufficient data in the terminal elimination phase following hemodialysis without increasing the total number of collected samples. At each time point for plasma collection, participants had vital signs and a 12-lead electrocardiogram monitored. For every participant, hemodialysis began at 18 h post-dose, and blood samples were collected at 20 (mid-way through hemodialysis) and 22 h post-dose. The participants were standardized to receive a 4 h session of hemodialysis using a polysulfone filter (Optiflux 200, Fresenius Medical Care) at their individualized usual blood and dialysate flow rates and under physician monitoring.

### Sample handling and analysis

After sample collection, samples were centrifuged at 3000 rpm for 10 min at 4 °C. The plasma was removed and stored in duplicate at −80 °C until sent for assay in batch. Plasma samples were shipped on dry ice to Intertek Pharmaceutical Services (El Dorado, CA). Ranolazine, and its metabolites [CVT-2512, CVT-2514 and CVT-2738] and an internal standard D3-Ranolazine were extracted from 100 mcL of human plasma (K2EDTA) by protein precipitation extraction using 90:10 acetonitrile:methanol. The supernatant was then diluted and analyzed by liquid chromatography atmospheric pressure with tandem mass spectrometry. Run times were approximately 9.5 min. The lower limit of quantitation (LLOQ) was 50.0 ng/mL for ranolazine and 10.0 ng/mL for CVT-2512, CVT-2514 and CVT-2738. The upper limit of quantitation was 10,000 ng/mL for ranolazine and 2000 ng/mL for CVT-2512, CVT-2514 and CVT-2738. The coefficients of variation for ranolazine, CVT 2512, CVT-2514, and CVT-2738 were 1.12–3.39%, 1.6–7.74%, 4.88–8.87% and 3.97–9.38%, respectively.

### Data analysis

Descriptive statistics were used to report subject demographics. The plasma concentration (Cp) data were plotted against time off and on hemodialysis for each patient. Non-compartmental analysis for the extravascular administration of a single 500 mg dose (participants 1–3) and a single 1000 mg dose (participants 4–8) was used to estimate the pharmacokinetic parameters for each patient using Winnonlin (Version 5.3). The mean (± SD) for the pharmacokinetic parameters off-hemodialysis is reported, excluding data from participant 3 whose ranolazine concentrations were below the LLOQ after 15 h. The extent of drug absorbed was assessed using the *AUC* by applying the linear trapezoidal rule. The observed *C_max_* and the time taken to reach the maximum observed drug concentration (*T_max_*) were based on actual observations. The apparent volume of distribution (*Vz/F*) and the apparent clearance (*Cl/F*) were normalized to patient body weight. The percent reduction ratio was calculated comparing the plasma concentrations post-hemodialysis (*t* = 23 h) to the concentration pre-hemodialysis (*t* = 18 h) using the formula: Percent reduction ratio = (Cp_18 h_–Cp_23 h_)/Cp_18 h._

Statistical analysis was done using unpaired *T* test in SPSS with the significance set at *p* < .05

## Results

Seventeen patients receiving maintenance hemodialysis were consented and screened for participation. Five patients did not meet inclusion criteria due to QTc interval length. Three patients voluntarily withdrew consent due to difficulty placing intravenous access, and one was admitted into the hospital prior to their scheduled participation. In total, 8 patients were enrolled and completed the study. The demographics of the participants are listed in Table 1. The study participants continued all their usual maintenance medications during the study period. Maintenance medications were confirmed by the medical research team to not include any major P-gp inhibitors, CYP3A4 inducers, or CYP3A4 inhibitors. They were instructed to not consume any major P-gp inhibitors, CYP3A4 inducers or CYP3A4 inhibitors.

**Table 1.** Participant demographics.

Characteristic Median (range)	Received 500 mg of ranolazine (*N* = 3)	Received 1000 mg of ranolazine (*N* = 5)
Gender	2 M, 1 F	3 M, 2 F
Race	2 Caucasian, 1 Latin American	3 Caucasian, 2 African American
Age (years)	32 (30–53)	33 (20–63)
Weight (kg)	54 (48–68)	86 (55–93.5)
Hemoglobin (g/dL)	12.2 (10.1–12.3)	10.7 (10.1–11.6)
Albumin (g/dL)	4.7 (4.2–4.8)	4.1 (2.7–4.8)
Baseline QTc Interval (msec)	429 (416–439)	455 (412–467)

Six of the eight participants received hemodialysis using an arteriovenous fistula or graft and two received hemodialysis via tunneled catheter. Upon completion of hemodialysis, all participants had a urea reduction ratio of ≥70% which would be approximately equivalent to a single pool kt/V_urea_ of ≥1.4. For all 8 participants completing the study, no substantial change in vital signs, including heart rate and blood pressure, were appreciated. In addition, no substantial increase or decrease in the QTc interval was appreciated throughout the study period. The only electrocardiogram finding was for the hyperkalemia mentioned below, but was determined to be unrelated to the administration of ranolazine. None of the participants reported any other adverse effect associated with ranolazine.

**Figure 1.**
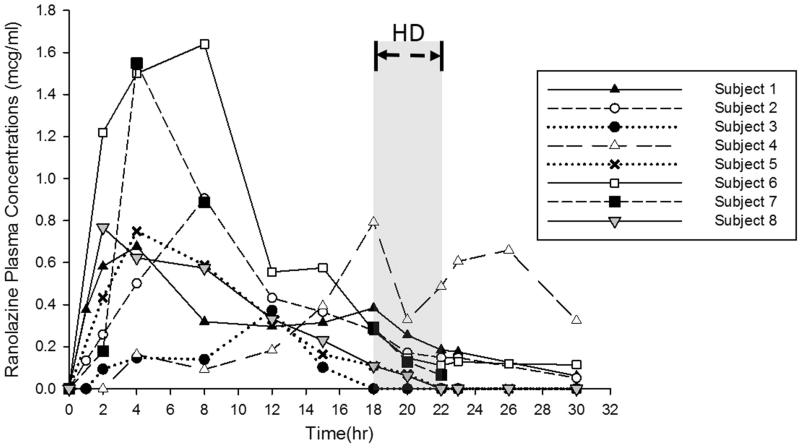
Ranolazine plasma concentration-time profiles in patients receiving either 500 mg (Subjects 1–3) or 1000 mg (Subjects 4–8) doses. HD designates the period during which hemodialysis occurred.

The three participants who completed the study prior to the interim analysis were administered a single ranolazine 500 mg tablet with lunch 18 h prior to the start of hemodialysis. A total of 13 samples was collected from each of the three participants prior to the interim analysis. A non-compartmental analysis was used to estimate the pharmacokinetic parameters for each subject. The ranolazine plasma concentration time curves are presented in Figure 1. For the 500 mg dose, the ranolazine half-life was calculated using the 23 h and 30 h samples for 2 participants. For the 3rd participant, the ranolazine half-life was calculated using the samples collected at 12 and 15 h as ranolazine concentrations were below the limit of quantification after 15 h. The mean (± SD) observed maximum ranolazine concentration was 0.65 (± 0.27) mcg/mL with an off-hemodialysis half-life of 3.6 ± 1.73 h. After the interim analysis, all 5 participants who completed the study received two ranolazine 500 mg tablets fasting 2 h before administration and 2 h after administration to minimize the contribution of food to the variability in absorption. All 13 samples were collected from 4 of the 5 participants receiving the 1000 mg dose. One participant had only 8 of 13 samples collected following electrocardiogram changes from an elevated potassium concentration attributed to dietary indiscretion. That participant underwent urgent hemodialysis at 10 h into the study. The mean ± SD observed maximum ranolazine plasma concentration was 1.18 ± 0.48 mcg/mL. The ranolazine half-life was calculated using the last 3–4 points with an off-hemodialysis half-life of 3.9 ± 0.55 h. The ranolazine pharmacokinetic parameters off-hemodialysis are listed in Table 2. When comparing the data for the 500 mg dose and the 1000 mg dose, despite the considerable difference in the pharmacokinetic parameters, it is noteworthy to highlight that our estimation of the half-life was quite comparable.

**Table 2.** Off-hemodialysis ranolazine pharmacokinetic parameters for patients receiving 500 mg or 1000 mg of oral ranolazine doses.

Parameter Mean ± SD	Received 500 mg of ranolazine (*N* = 3)	Received 1000 mg of ranolazine (*N* = 4)^a^
Maximum concentration (mcg/mL)	0.65 ± 0.27	1.18 ± 0.48
Time to maximum concentration (hr)	8 ± 4	4.5 ± 2.5
Elimination phase apparent half-life (hr)	3.6 ± 1.73	3.9 ± 0.55
Area under the curve 0–18 hr (hr* mcg/mL)	6.17 ± 3.2	10.33 ± 4.87
Apparent volume of distribution (L/kg)	14.37 ± 3.2^b^	7.3 ± 1.5
Apparent off-hemodialysis clearance (L/hr/kg)	0.66 ± 0.23^b^	1.34 ± 0.41

a Excludes the data from 1 participant who had inconsistent plasma ranolazine results.

b Excludes the data from 1 participant who had ranolazine concentrations below the LLOQ after 15 h.

**Figure 2.**
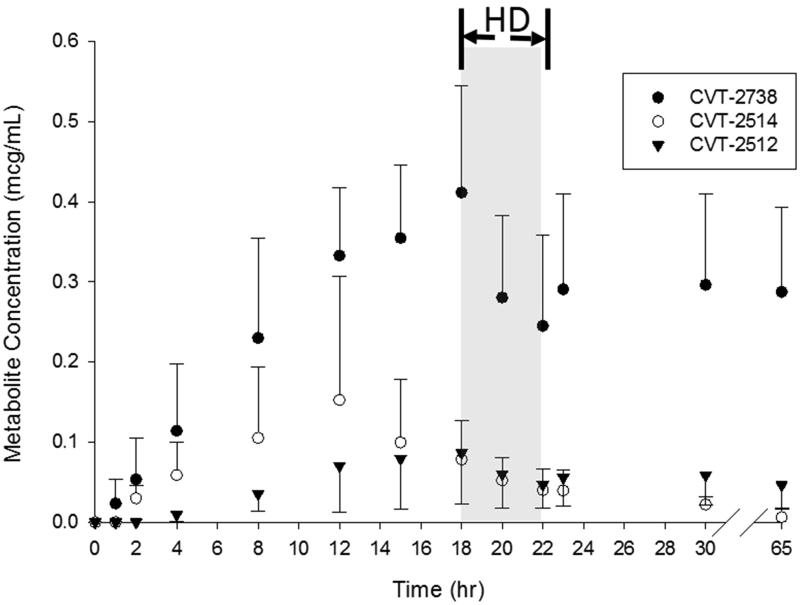
Plasma concentration-time profiles for the three major metabolites of ranolazine in patients receiving a 500 mg oral dose of ranolazine. Data is presented as mean ± standard deviation. HD designates the period during which hemodialysis occurred.

**Figure 3.**
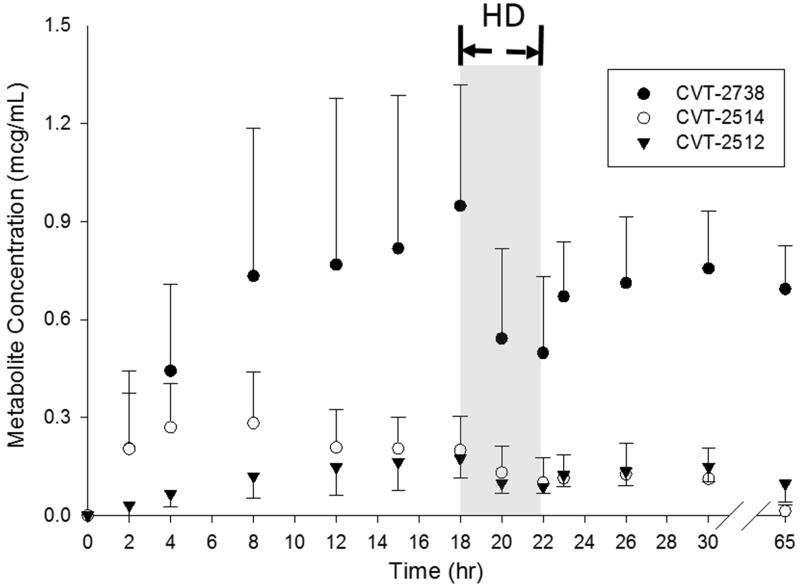
Plasma concentration-time profiles for the three major metabolites of ranolazine receiving a 1000 mg oral dose of ranolazine. Data is presented as mean ± standard deviation. HD designates the period during which hemodialysis occurred.

Figures 2 and 3 show the plasma concentration- time profiles for the three major metabolites of ranolazine. As expected, an increase in the plasma concentrations of the metabolites was accompanied by a decrease in the plasma concentrations of the parent drug with CVT-2738 showing the greatest AUC. Overall, the CVT-2738 metabolite was produced in higher quantities than either CVT-2512 or CVT-2514. The average *AUC* (0–12h) for each of the three metabolites was lower than the corresponding ones reported for participants with severe renal impairment [11].

The mean ± SD percent dialysis reduction ratio for the ranolazine 500 mg was 52.3 ± 8.1%, and the percent reduction ratios for metabolites CVT-2512, CVT-2514, and CVT-2738 were 29.1 ± 12.6%, 38.1 ± 33.1%, 31.1 ± 8.3%, respectively. The percent reduction ratios for the ranolazine 1000 mg dose was 69.2 ± 37.6%, excluding the patient who did not have all 13 samples collected, and the reduction ratios for metabolites CVT-2512, CVT-2514, and CVT-2738 were 26.3 ± 2.6%, 41 ± 10.6%, and 17.4 ± 21.0%, respectively, which are comparable to the estimations obtained in 500 mg dose analysis. Evidence for a post-hemodialysis rebound phenomenon could be seen with ranolazine and its metabolites in some patients. Ranolazine and metabolite concentrations obtained one hour after hemodialysis ended (Cp at 23 h) tended to be higher than the observed Cp values immediately post hemodialysis (Cp at 22 h) in those patients.

## Discussion

To date, no previously published study has evaluated the oral ranolazine pharmacokinetics or extent of removal during hemodialysis in patients receiving maintenance hemodialysis. *This study is the first to attempt to estimate ranolazine pharmacokinetics following a single dose in hemodialysis patients.* Oral ranolazine pharmacokinetics after multiple doses of 500 mg in patients with varying degrees of kidney dysfunction has been reported [11]. In that study, participants were administered the extended release drug formulation; which we administered in our study, and were divided into 4 groups based on their calculated creatinine clearance (CrCl) with severe renal impairment defined as a CrCl < 30 mL/min, but not dialysis dependent. Jerling and Abdallah showed that the apparent half-life was not prolonged with the increasing degree of renal impairment. With the flip-flop pattern displayed by the extended release formulation, the absorption phase is prolonged and the elimination phase apparent half-life is shorter than the absorption half-life. Hence, changes in the true elimination do not substantially alter the observed apparent half-life. As the severe renal impairment group is the closest comparator to the participants in our study, we used this group as our comparator in our analysis. The mean half-lives with the 500 mg and 1000 mg dose (3.6 and 3.9 h, respectively) were not significantly different (*p* < .05) from each other and are similar to the 4.6 h mean half-life published for participants with severe renal impairment [11]. Prior to the interim analysis in this study, the time to maximum concentration varied greatly, and each participant had a different time to maximum concentration (4, 8, and 12 h). There were no protocol variations or other external factors that would account for the variability. After the interim analysis, participants fasted 2 h before and 2 h after the dose of ranolazine to help control for absorption variability, but *T_max_* continued to vary with each participant (from 2 to 18 h). Large absorption variability is documented in the package insert [9], and is consistent with the findings discussed herein. After receiving the 500 mg or 1000 mg dose of oral ranolazine, the mean *C_max_* was 0.66 mcg/mL or 1.18 mcg/mL, respectively. When compared to the published mean *C_max_* at steady state (2.4 mcg/mL) seen in participants with severe renal impairment receiving a dose of 500 mg [11], our participants’ mean *C_max_* (1.2 mcg/mL) was half of that reported previously. Similarly, our ranolazine *AUC*_0–18_ (10.3 h*mcg/mL) was less than half of the ranolazine *AUC*_0–12_ as reported by Jerling and Abdallah for participants with severe renal impairment. Together, the substantially lower *C_max_* and *AUC* values highlight the extent of decrease in drug bioavailability in patients on hemodialysis. In this study, the CVT-2738 metabolite was produced overall in higher quantities than either CVT-2512 or CVT-2514. When comparing the metabolites CVT-2512, CVT-2514, and CVT-2738, we saw similar results as observed with the parent ranolazine compound. Reduction ratios of ranolazine and metabolites indicate that hemodialysis contributes to the clearance of these compounds to differing extents.

This pilot study has limitations. We collected our samples from a relatively small population of 8 participants of which 3 received a 500 mg dose and 5 received a 1000 mg dose of ranolazine. We collected a limited number of samples from each participant for pharmacokinetic analysis due to the propensity of dialysis patients to develop anemia. We did not calculate mass balance or collect dialysate samples during the hemodialysis sessions since the assay’s LLOQ did not allow for measurement of ranolazine or metabolite concentrations in the dialysate. Ranolazine is extensively metabolized by the liver CYP3A and 2D6 enzymes and hepatic CYP3A4 activity possibly is acutely improved by conventional hemodialysis [12,13]. We did not assess the effect of uremic toxin removal by hemodialysis on restoration of metabolic CYP activity.

Based on our pharmacokinetic results of an oral dose of ranolazine 500 mg and 1000 mg, neither dose can be recommended as the initial starting dose for this population. After one dose of 500 mg or 1000 mg, the average maximum concentrations were similar to the healthy volunteers in the Jerling and Abdullah study [11] but were slightly less than half of the steady state maximum concentrations reported in the package insert from healthy volunteers and those in the Jerling and Abdullah study in the severe renal impairment group [9,11] In addition, large variability was seen amongst the study participants in terms of the percent reduction ratio by hemodialysis, *T_max_*, *C_max_*, *Vz/F*, and *AUC*_0–18_.

Despite the variability observed in our study and given the scarcity of reported studies in the literature, the data presented herein provides useful guidance to clinicians as they tackle the complexity of dosing ranolazine to patients on maintenance hemodialysis. Ranolazine’s prescribing information lists dosing recommendations as 500 mg twice daily and increase to 1000 mg twice daily as needed with or without meals. Despite specific claims of absence of any food effect on the *C_max_* and *AUC* of ranolazine, the drug was reported to have a high degree of absorption for the extended release formulation throughout the gastrointestinal tract and absorption extends beyond 12 h post dose [10]. Our findings suggest that a dose higher than 1000 mg may be necessary to achieve therapeutic plasma levels. In designing future clinical trials, investigators are encouraged to consider fasting state and to tailor the sampling scheme to capture times of highest variability as reflected in Figure 1. Further large scale prospective multiple-dose studies are necessary in patients undergoing maintenance hemodialysis before a dosing regimen can be tailored for this population.
